# The association between moderate-to-vigorous physical activity and health-related quality of life in Chinese adolescents: the mediating roles of emotional intelligence and perceived stress

**DOI:** 10.3389/fpsyg.2024.1477018

**Published:** 2024-12-02

**Authors:** Qianyuan Li, Li Li, Chuchu Li, Huilin Wang

**Affiliations:** ^1^School of Physical Education, Hunan University of Science and Technology, Xiangtan, China; ^2^Moray House School of Education and Sport, The University of Edinburgh, Edinburgh, United Kingdom

**Keywords:** adolescents, moderate-to-vigorous physical activity, emotional intelligence, perceived stress, health-related quality of life

## Abstract

**Introduction:**

Chinese adolescents are facing tremendous academic pressure and challenges brought about by changes in the social environment, which pose a serious threat to their health-related quality of life (HRQOL). This cross-sectional survey uses convenience and snowball sampling to explore the relationship between adolescents’ HRQOL and their participation in moderate-to-vigorous physical activity (MVPA).

**Methods:**

This study subjects include 440 adolescents aged 12 to 18, from four youth training centers and three schools in the central regions of Hunan, Hubei, and Henan provinces, chosen as representative samples for this study. We used AMOS v.26 to construct a structural equation model for data analysis and hypothesis testing.

**Results:**

The results indicated that active participation in MVPA helps reduce adolescents’ perceived stress and enhances their emotional intelligence and HRQOL levels. Specifically, MVPA weakens adolescents’ perceived stress through the mediating role of emotional intelligence (coefficient = −0.148, *p* < 0.001), and perceived stress also mediates the relationship between emotional intelligence and HRQOL (coefficient = 0.165, *p* < 0.001). Furthermore, the positive impact of MVPA on HRQOL is mediated by emotional intelligence and perceived stress (coefficient = 0.363, *p* < 0.001). The explanatory power of this study is *R*^2^ = 0.50.

**Conclusion:**

The study results indicate that MVPA has a positive impact on the HRQOL levels of Chinese adolescents. Physical activity, especially moderate-to-vigorous intensity exercise, should be considered a strategic approach to maintaining HRQOL among adolescents. Society, schools, and families should create an environment conducive to physical exercise to support adolescents in engaging in physical activities and developing a healthy lifestyle.

## 1 Introduction

Adolescence is a critical period between childhood and adulthood, regarded as an essential phase for addressing past health issues and laying the foundation for future health status (Boraita et al., 2020). Establishing some healthy lifestyle patterns in adulthood also benefits from the health opportunities encountered during this period (Sawyer et al., 2012). Moreover, the quality of life in adulthood is influenced by the continuation of lifestyle habits formed during adolescence (Bisegger et al., 2005; Frisén, 2007). However, this period is also a vulnerable time when adolescents are more prone to physical and mental health disorders. This heightened vulnerability is due to increased neural plasticity, more intense emotional responses, and greater sensitivity to external environmental changes, making them more susceptible to influences from society, family, and peers (Lee et al., 2014). At the same time, adolescents also undergo involuntary physical changes as their bodies mature, along with hormonal surges that lead to shifts in personality (Best and Ban, 2021). The variations in the timing of development and the comparison of physical appearance with peers can result in dissatisfaction with body image, social anxiety, and depression (Soares Filho et al., 2021; Rapee et al., 2022), which, in turn, affect their social skills, socialization process, and even mental health levels (Pfeifer and Allen, 2021). Consequently, the formation of bad habits such as smoking, alcohol abuse, and prolonged sedentary behavior often occurs during adolescence (Jackson et al., 2012), potentially impacting their future lifestyle choices and indirectly increasing the burden on public healthcare services (Moore et al., 2018).

Various data and studies indicate that complex social environmental factors, such as the rapid proliferation of social media platforms in the 21st century (e.g., Facebook, Instagram, and Weibo) (Lam and Peng, 2010), deteriorating family environments (including post-war single-parent families, domestic violence, and community violence incidents) (Panter-Brick et al., 2009), the impact of the global financial crisis (Parker et al., 2016), and the COVID-19 pandemic (Magson et al., 2020), have collectively had varying degrees of negative impact on adolescents’ HRQOL. Evidently, the massive cultural, economic, and social changes have posed unprecedented challenges to this generation of adolescents, potentially making them the most at-risk generation for mental health and HRQOL issues in history (Patton et al., 2016). HRQOL is essential for assessing adolescents’ quality of life (González-Cabrera et al., 2021), offering insights into their perceptions, understanding their needs, and identifying early barriers to well-being (Haraldstad et al., 2011). At the same time, adolescents with poor HRQOL are likely to face challenges entering a healthy developmental state, hindering their growth into healthy adults (Bermejo-Cantarero et al., 2021). However, in previous HRQOL studies, the focus on adolescents has been significantly lower than on adults, and our understanding of the internal mechanisms of the interaction between HRQOL and other variables is still limited.

Current research on adolescents’ HRQOL mainly examines factors such as adolescent bullying (Dubey et al., 2022), adverse and positive childhood experiences (Luo et al., 2024), the influence of parents, teachers, and peers (Tilga et al., 2021), and issues like obesity and severe overweight (van de Pas et al., 2023). Although some studies have noted that engaging in physical activities that inherently appeal to adolescents, particularly at higher levels of activity (Wu et al., 2017; Zhou et al., 2024), can not only improve physical fitness but also reduce appearance anxiety and improve HRQOL levels (Bermejo-Cantarero et al., 2021; Jiménez Boraita et al., 2024), there is currently a lack of research on how adolescents’ participation in MVPA affects their HRQOL. Our understanding of the internal mechanisms of how this behavioral level affects the cognitive level remains insufficient. Additionally, China has a vast population of adolescents (Qin et al., 2021) and a unique social culture and education system, which results in greater academic pressure and sharper HRQOL issues for Chinese adolescents (Zeng et al., 2022). Focusing on Chinese adolescents offers specific insights into issues within the Chinese social environment. This study uniquely explores the mechanisms linking MVPA and HRQOL, emphasizing the mediating roles of perceived stress and emotional intelligence. Perceived stress addresses the prevalent stress in Chinese adolescents, while emotional intelligence aids in better emotional regulation. In summary, building on previous HRQOL research, this study aims to: (1) analyze the relationships between MVPA, perceived stress, emotional intelligence, and HRQOL; (2) explore the mediating roles of cognitive factors (perceived stress and emotional intelligence) between MVPA and HRQOL; and (3) communicate the research findings to adolescents’ families, communities, and schools, and provide effective recommendations.

This study focuses on the HRQOL of adolescents, considering the impact of behavior on cognition, and explores the internal mechanisms between MVPA and HRQOL. Actively participating in MVPA, such as ball sports, track and field, gymnastics, water or ice sports, traditional Chinese sports, and emerging sports, as recommended in the new Chinese curriculum, gives adolescents more opportunities for social interaction and emotional development, thereby boosting their emotional intelligence (Gu, 2022). Increased emotional intelligence further improves adolescents’ abilities to regulate their emotions and cope with stress, anxiety, depression, and other negative emotional issues, thereby improving their HRQOL. The results indicate that emotional intelligence positively mediates the relationship between MVPA and HRQOL, while perceived stress negatively mediates it. Adolescents engaging in MVPA tend to have higher emotional intelligence, lower perceived stress, and better HRQOL. Understanding this relationship deepens research on MVPA and HRQOL and offers theoretical guidance for protecting adolescents’ HRQOL, encouraging families, communities, and schools to promote MVPA.

## 2 Literature review and hypothesis development

### 2.1 Variable definitions

#### 2.1.1 MVPA

Research consistently shows that physical activity is essential for a healthy lifestyle. It induces beneficial physiological changes, reduces the negative impact of stress on health, and supports mental well-being (Gerber et al., 2017). In fact, the impact of different exercise intensities on adolescents’ mental health varies (Pascoe et al., 2020). While international standards suggest that participating in 150 min of moderate to vigorous physical activity per week can reduce the risk of chronic diseases by nearly one-third later in life (Mahindru et al., 2023), for adolescents in their developmental stage, actively following the ‘24-h movement guidelines’ (i.e., achieving 3 h of physical activity per day, with at least one hour being MVPA) within their psychological and physical capacity can lead to more positive mental health benefits (Tremblay et al., 2016; Tapia-Serrano et al., 2022). MVPA specifically refers to physical activities that range from moderate to vigorous intensity levels (Nader et al., 2008). Existing literature has reached a consensus that regular participation in physical activities, especially MVPA during adolescence, is particularly effective in enhancing cardiovascular health (Knaeps et al., 2016), improving type 2 diabetes (Blomster et al., 2013), and addressing psychological health issues such as low self-esteem and depression (Hollis et al., 2017). Additionally, engaging in MVPA during the developmental stage of adolescence can improve suboptimal health conditions and effectively lay the foundation for preventing chronic diseases in adulthood (e.g., heart disease) (Skrede et al., 2017). Due to the more pronounced neurological changes induced by MVPA, its positive effects on adolescents’ cognitive abilities and mental health are also significant (Nakagawa et al., 2020). This study explores the concept of MVPA based on the new curriculum activities in the daily lives of Chinese adolescents (Gu, 2022).

#### 2.1.2 Emotional intelligence

Salovey and Mayer (1990) first introduced the concept of emotional intelligence, defining it as a subset of social intelligence. It involves recognizing and managing one’s own and others’ emotions to guide thinking and behavior. Over the past few decades, the discussion on emotional intelligence has been ongoing, with the definition of emotional intelligence becoming a research hotspot. Current research on emotional intelligence mainly falls into three categories: (1) Ability-based models define emotional intelligence as a skill for integrating and processing personal emotions and external information (Estrada et al., 2021). (2) Competency models, where Nelis et al. (2009) view emotional intelligence as a collection of various abilities, including self-control, emotional regulation, and self-regulation. (3) Trait and mixed models view emotional intelligence as a stable set of personality traits, encompassing social–emotional abilities, self-efficacy, and cognitive aspects (Petrides et al., 2016). This study follows Law et al. (2004) definition, which describes emotional intelligence as the ability to understand, enhance, and manage one’s emotions while integrating and processing the emotions of others. Since adolescents experience greater uncertainty and competition during physical activity and must adapt to the intense emotional fluctuations it brings, this can effectively enhance their self-emotional management abilities (Wang et al., 2020; Zhang et al., 2022). Additionally, adolescents in a sporting environment are exposed to a sense of teamwork and social support, and they actively or passively gain more opportunities for interpersonal communication, which helps improve their ability to perceive and regulate others’ emotions (Wang et al., 2022).

#### 2.1.3 Perceived stress

Stress has been a key focus in academic research and is vital in health sciences. It is viewed as a stimulus, a response, or an interaction between the individual and their environment (Schiffrin and Nelson, 2010; Siqueira Reis et al., 2010). Stress is associated with various physical and mental health diseases, including well-being, cancer, and coronary heart disease (Schiffrin and Nelson, 2010; Richardson et al., 2012). Perceived stress refers to the subjective level of stress an individual feels. Different individuals have varying perceptions of stress, making perceived stress a highly subjective concept related to current emotions and environmental factors (Piqueras et al., 2011; Ruiz-Aranda et al., 2014). Adolescents’ perceived stress is particularly unstable due to the fluctuating hormone levels during puberty, leading to significant emotional swings (Salovey et al., 2002). Additionally, perceived stress during adolescence increases with age and is influenced by factors such as gender, interpersonal relationships, and personality traits (Hampel and Petermann, 2006). The accumulation of stress, coupled with the immature state of adolescents, can easily lead to adverse reactions such as obesity and mental illnesses (van Jaarsveld et al., 2009). However, previous research has found that participating in physical activities can enhance adolescents’ tolerance and adaptability to distress, thereby helping them alleviate perceived stress levels in stressful environments (Wright et al., 2023). Additionally, through physical activities, adolescents can develop a positive sense of self-awareness and control, which helps divert their attention and negative thoughts about known stressors, thus reducing the amount of stress they perceive (Mikkelsen et al., 2017; Wright et al., 2021). Even short-term, moderate to low-intensity aerobic exercise can effectively lower overall perceived stress levels (Herbert et al., 2020).

#### 2.1.4 HQROL

Quality of life is a broad and widely discussed concept encompassing all aspects of an individual’s life (Ware, 2003). HRQOL specifically refers to the aspects of quality of life that affect an individual’s health (Torrance, 1987). HRQOL was first applied in the medical field to measure the outcomes of traditional clinical treatments, where besides mortality and morbidity, HRQOL was used as an endpoint to evaluate the effectiveness and usefulness of a patient’s life (Crosby et al., 2003). HRQOL is defined as an individual’s subjective assessment of their physical health, psychological well-being, and the health of their social relationships (Gomes et al., 2020).

In fact, physical activities conducive to health, even in non-specific domains such as commuting and household chores, positively contribute to HRQOL across all age groups (Jurakić et al., 2010), with subjective self-assessments and objective accelerometer measurements both showing strong outcomes (Wright et al., 2023). Conversely, patients experiencing physical limitations due to injuries or chronic illness often see significant declines in HRQOL (Pinquart, 2020). Mental health issues also seriously impact HRQOL levels, with individuals experiencing anxiety, depression, and stress typically scoring lower on HRQOL (Manh Than et al., 2020). Furthermore, studies on healthy aging have found that older adults with low communication frequency and limited social interaction factors—such as social isolation, lack of social support, and feelings of loneliness—also experience diminished HRQOL (de Belvis et al., 2008; Freak-Poli et al., 2022). In this study, HRQOL refers to the overall evaluation of adolescents’ health in terms of physical, psychological, and social interactions.

### 2.2 Hypotheses

#### 2.2.1 MVPA, emotional intelligence, perceived stress, and HRQOL

Research increasingly shows that adolescents develop emotional perception and control mechanisms during physical activity and sports training (Acebes-Sánchez et al., 2019; Wang et al., 2020). On one hand, the real social experiences from peer cooperation or intense competition in vigorous physical activities can enhance adolescents’ empathy and ability to understand and respond to others’ emotions (Ubago-Jiménez et al., 2019). On the other hand, engaging in MVPA in moderation not only brings various physiological benefits to adolescents but also enhances their ability to acquire social–emotional skills and self-regulation abilities in the cognitive domain (Ubago-Jiménez et al., 2021). Additionally, studies have found that contemporary adolescents are adept at alleviating personal emotional distress through resources they can actively access (e.g., joining sports teams) (Martorell-Poveda et al., 2015), and their levels of emotional intelligence (especially in emotional attention and repair) increase with the intensity and frequency of their physical activity participation (Acebes-Sánchez et al., 2019).

In fact, individual resilience in adolescents is a crucial force that regulates their adaptive changes when facing negative environments and stressful situations (Piekarska, 2020). Liu et al. (2017) multisystem resilience model identifies emotional intelligence as a core component, essential to adolescents’ overall resilience. Furthermore, adolescents are constrained by their limited life experience, which often makes it difficult for them to cope with changes in various life events, frequently viewing significant life changes as sources of perceived stress (Cejudo et al., 2018). However, a meta-analysis by Sánchez-Álvarez et al. (2016) found that effective emotional strategies can mitigate the negative emotions arising from sudden external events, enhancing individuals’ positive expectations for future events and their current subjective well-being. Additionally, the emotional intelligence improved through physical exercise can, in conjunction with physical exercise, help adolescents alleviate their feelings of frustration under stress and assist them in making reasonable behavioral decisions in various challenging situations (Puertas Molero et al., 2017).

Early research indicates that perceived stress severely damages adolescents’ internal psychological resources and is a major factor leading to mental health disorders (Corell et al., 2021). If adolescents cannot positively confront the burdens and unpredictable circumstances in life, these will accumulate and transform into a stress-filled living environment (Grant et al., 2004). When adolescents are in high-perceived stress environments, they are more likely to experience anxiety, depression, and other negative psychological states, thereby lowering their HRQOL levels (Racic et al., 2017). Moreover, excessive accumulation of perceived stress can lead to mental disorders in adolescents, and these adverse mental states can persist into adulthood and beyond, severely damaging their HRQOL throughout their lifespan (Lindholdt et al., 2021). In a study by Kaur and Agarwal (2022) focusing on young adults with an average age of around 20, high perceived stress was found to be significantly and consistently negatively correlated with physical fitness and HRQOL, with perceived stress explaining nearly 40% of the overall differences in HRQOL. Based on the relevant theories and research, we propose the following hypotheses:

*H1:* Emotional intelligence mediates the relationship between MVPA and perceived stress.

*H2:* Perceived stress mediates the relationship between emotional intelligence and HRQOL.

#### 2.2.2 The mediating effects

Adolescents experience rapid and highly sensitive physiological and psychological development. Increasing academic pressure and social anxiety can impact their mental health (Reverte-Villarroya et al., 2021). Psychological disorders often begin in adolescence, particularly with the onset of depression caused by stress and anxiety, typically occurring before the age of 20. This condition not only damages the HRQOL of young people but also hinders their future prospects by impeding their entry into the workforce (Dhillon et al., 2020). Fortunately, numerous studies have shown that physical exercise, especially MVPA, can enhance adolescents’ vitality, improve their cognitive abilities, reduce perceived stress, and increase their social opportunities and skills with friends, thereby enhancing their emotional intelligence (Hollis et al., 2017; Ubago-Jiménez et al., 2019; Nakagawa et al., 2020; Nagata et al., 2022). MVPA stimulates physiological responses like brain-derived neurotrophic factor, dopamine, and endorphins, aiding in rapid adaptation to these physiological mechanisms (Chang et al., 2012; Skrede et al., 2017). Additionally, MVPA can improve mood, maintain or enhance physical appearance, and validate personal fitness, all of which positively affect adolescents’ psychological cognition and mental state (Parker et al., 2022).

Current research on the mediating effects of adolescents’ emotional intelligence and perceived stress mainly addresses well-being (Sanchez-Ruiz et al., 2021), self-assessed health status (Wang et al., 2023), decreased immune function (Lueke and Assar, 2024), and mental health (Malinauskas and Malinauskiene, 2020). Furthermore, some studies have found an inhibitory relationship between adolescents’ MVPA and perceived stress and various psychological disorders such as anxiety and depression (Tajik et al., 2017). In fact, perceived stress is likely one of the most crucial factors reducing adolescents’ HRQOL (Mikkelsen et al., 2020). However, MVPA can enhance self-awareness, improve adolescents’ perceptions of self-worth, and increase self-esteem, thereby reducing perceived stress and fostering mental health (Hale et al., 2021). Additionally, evidence suggests that positive emotional intelligence plays a crucial role in children’s self-regulation of health-related behaviors and can impact the HRQOL of those with body image dissatisfaction (Pollatos et al., 2020). Based on the relevant theories and research, we propose the following hypothesis:

*H3:* Emotional intelligence and perceived stress mediate the relationship between MVPA and HRQOL.

**Figure 1:**
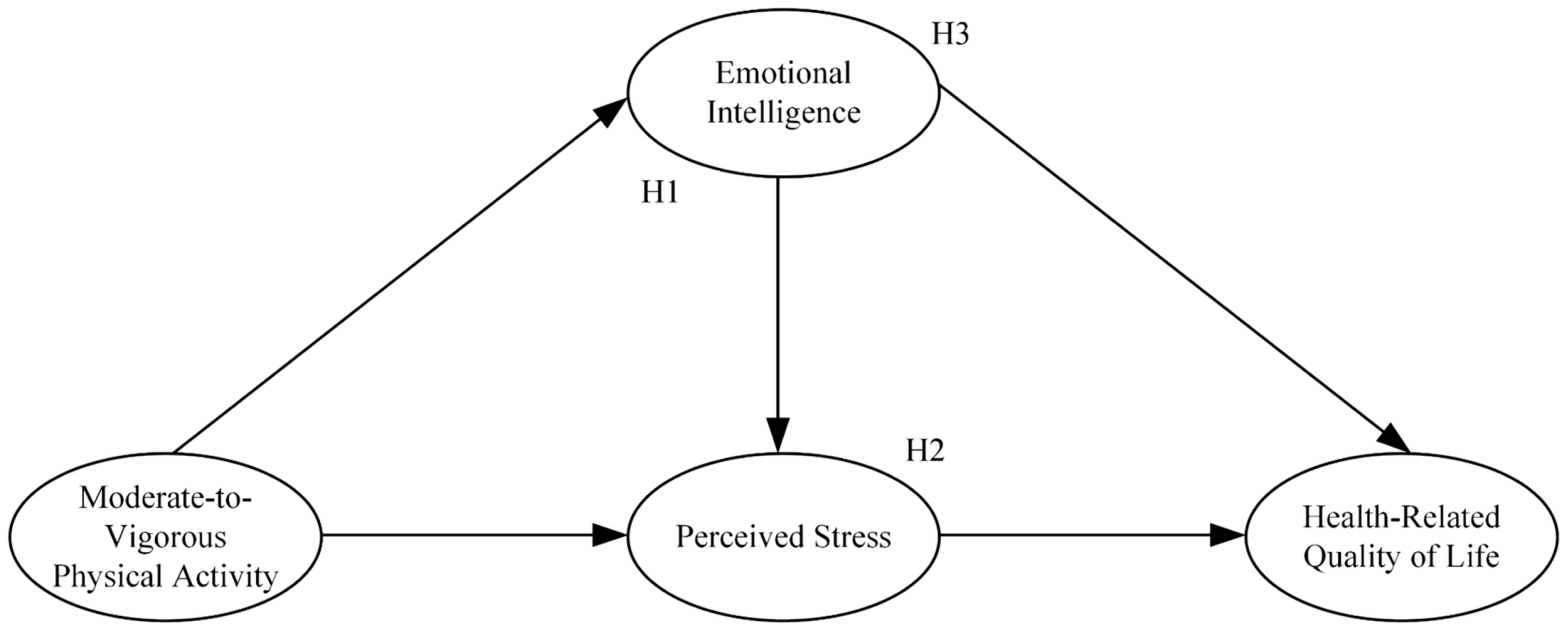
The conceptual model.

This study proposes three hypotheses: First, adolescents’ emotional intelligence mediates the relationship between MVPA and perceived stress. Second, adolescents’ perceived stress mediates the relationship between emotional intelligence and HRQOL. Lastly, emotional intelligence and perceived stress play a mediating role in the relationship between adolescents’ MVPA and HRQOL. An overview of all hypotheses is illustrated in Figure 1.

## 3 Methodology

### 3.1 Participants and procedure

This study used convenience sampling and snowball sampling techniques to recruit adolescents aged 12–18 for an online survey. To collect data, we contacted administrators of four after-school sports service training centers and three schools in the central regions of China (Hunan, Hubei, and Henan provinces). Based on the adolescent population in these three provinces and the sample size formula *n* = N/(N(*δ*)^2 + 1), the sample size for this study was calculated to be approximately 400 participants. Upon obtaining consent, we sent messages to the target group through social media platforms such as WeChat. These messages explained the purpose of the study and included an invitation to participate. If they replied with “1,” indicating their willingness to participate, we sent them a link to the survey tool (Wenjuanxing app). To express our gratitude, participants received a random reward ranging from 10 to 20 RMB upon completing the questionnaire. Additionally, we encouraged participants to share the survey link with their classmates, friends, or peers at after-school sports service training centers.

Participants recruited through the snowball method completed the questionnaire, and their responses were automatically sent back to the researchers via the Wenjuanxing app. Inclusion criteria required participants to be aged 12–18 with some sports experience (having at least one favorite sport and participating in sports at least once per week on average). The study had 482 participants. After excluding invalid questionnaires, 440 usable responses were obtained, resulting in an effective response rate of 91.2%.

Table 1 details the demographics of the 440 adolescent participants. Key findings include: (1) About 58% were aged 12 to 15 years. (2) The gender ratio was about 3:2, with more males than females. (3) A significant number of participants were involved in sports such as basketball, track and field, and badminton, accounting for about 50% of the total. (4) About 46% of the participants engaged in sports activities four to five times per week on average over the past year, aligning with the frequency of physical education classes and after-school sports services provided on school days in China.

**Table 1:** Demographic overview of survey participants (*N* = 440).

Profiles		n	%
Age	12–15	254	57.7
	16–18	186	42.3
Gender	Male	276	62.7
	Female	164	37.3
Sports activities	Basketball	96	21.8
	Athletics	70	15.9
	Badminton	64	14.5
	Soccer	44	10.0
	Table Tennis	34	7.7
	Volleyball	22	5.0
	Bodybuilding	19	4.3
	Swimming	16	3.6
	Other	75	17.0
Exercise frequency per week	1–3	142	32.3
	4–5	206	46.8
	6–7	92	20.9

### 3.2 Instruments

The questionnaire had five sections. The first section collected demographic information such as age, gender, types of sports activities, and weekly participation frequency over the past year. The remaining sections used established scales from renowned researchers to gather specific data in targeted areas. The second section integrated three items from the scale designed by Sallis et al. (1985) to measure the intensity of MVPA in the past week. An example item was: “In the past week, I actively engaged in various forms of high-intensity physical activities, including tasks such as lifting heavy objects and sports like jogging, soccer, and similar activities.” This scale had been previously used and validated (Tseng et al., 2020; Tsai et al., 2021). The third section used five items from the scale designed by Law et al. (2004) to assess participants’ emotional intelligence. An illustrative item was: “I am good at understanding my own emotions.” This scale had also been previously used and validated (Wang et al., 2023). The fourth section incorporated five items from the scale developed by Cohen et al. (1983) to evaluate participants’ perceived stress. An example item was: “In the past month, how often have you felt nervous and stressed?” This scale had been used and validated before (Tang et al., 2023). The final section adopted four items from the scale developed by Ravens-Sieberer et al. (2010) to obtain data on the HRQOL levels of the participating adolescents. An example item was: “Do you feel healthy?” This scale had been previously used and validated (Nik-Azin et al., 2014).

To assess the reliability of the revised questionnaire, we conducted a pilot test in Hunan Province, obtaining 96 valid responses. The results showed that all Cronbach’s alpha coefficients exceeded 0.8, confirming the researchers’ adjustments to the scales (la Du and Tanaka, 1989).

### 3.3 Data analysis

In this study, we used AMOS v.26 to create a structural equation model (SEM) to examine how adolescents improve their HRQOL through MVPA. We adopted a two-step modeling approach, starting with confirmatory factor analysis (CFA) of the measurement model to ensure the reliability and validity of the variables, including tests for internal consistency, construct validity, and discriminant validity. Following this, we evaluated the structural model’s fit indices and path coefficients to explore the relationships between variables and assess potential mediation effects. A significance level of *p* < 0.05 was established for all analyses to ensure statistical robustness and scientific validity of the findings.

To address common method variance (CMV) in self-reported data, we followed Mossholder et al. (1998) approach, comparing two models by assessing changes in degrees of freedom and chi-square values. The chi-square value for model one was 2360.902 with 119 degrees of freedom (*p* < 0.001), while model two had a chi-square value of 212.428 with 113 degrees of freedom (*p* < 0.001). These results confirm the equivalence of the two models, indicating no univariate structure and no CMV concerns in this study.

## 4 Results

### 4.1 Measurement model

The reliability and validity of the latent variables were evaluated through CFA using AMOS v.26. The CFA model demonstrated excellent fit indices: *χ*^2^/df = 1.880, GFI = 0.956, NFI = 0.964, TLI = 0.979, CFI = 0.983, and RMSEA = 0.041. These indices meet the established criteria, with *χ*^2^/df between 1 and 3, and GFI, NFI, TLI, and CFI all exceeding 0.9, while RMSEA is less than 0.08, indicating an excellent model fit.

Additionally, all variables showed Cronbach’s *α* values above 0.8, indicating strong internal consistency (see Table 2) (Fornell and Larcker, 1981). The average variance extracted (AVE) for each variable exceeded 0.5, and the composite reliability (CR) for each latent variable was over 0.8, confirming the model’s convergent validity. Factor loadings ranged from 0.680 to 0.878, underscoring robust construct validity. Discriminant validity was established as the square root of the AVE for each construct surpassed the correlations between constructs (see Table 3).

**Table 2:** Reliability and validity analysis.

Items	Loadings	Cronbach’s *α*	CR	AVE
Moderate-to-vigorous Physical activity (MVPA)		0.899	0.899	0.749
MVPA1	0.878			
MVPA2	0.860			
MVPA3	0.858			
Emotional intelligence (EI)		0.920	0.920	0.698
EI1	0.806			
EI2	0.864			
EI3	0.855			
EI4	0.817			
EI5	0.834			
Perceived stress (PS)		0.833	0.833	0.500
PS1	0.680			
PS2	0.703			
PS3	0.705			
PS4	0.709			
PS5	0.738			
Health-related quality of life (HRQOL)		0.908	0.908	0.711
HRQOL1	0.865			
HRQOL2	0.857			
HRQOL3	0.810			
HRQOL4	0.841			

**Table 3:** Pearson correlation.

Construct	MVPA	EI	PS	HRQOL
MVPA	(0.865)			
EI	0.352 **	(0.835)		
PS	−0.391 **	−0.444 **	(0.707)	
HRQOL	0.523 **	0.543 **	−0.530 **	(0.843)

On the diagonals, the square root of the AVE is located, while the off-diagonals represent Pearson’s corrections of contrasts. **p < 0.01.

### 4.2 Structural model

After assessing the reliability and validity of the measurement model, the study examined the structural model using AMOS v.26 to test the hypotheses. The CFA results, based on 5,000 bootstrap samples, met the recommended criteria (*χ*^2^/df = 2.354, GFI = 0.944, NFI = 0.955, TLI = 0.968, CFI = 0.973, RMSEA = 0.050), indicating an excellent fit between the model and the empirical data. Pearson correlation results in Table 3 support the established relationships between variables.

**Figure 2:**
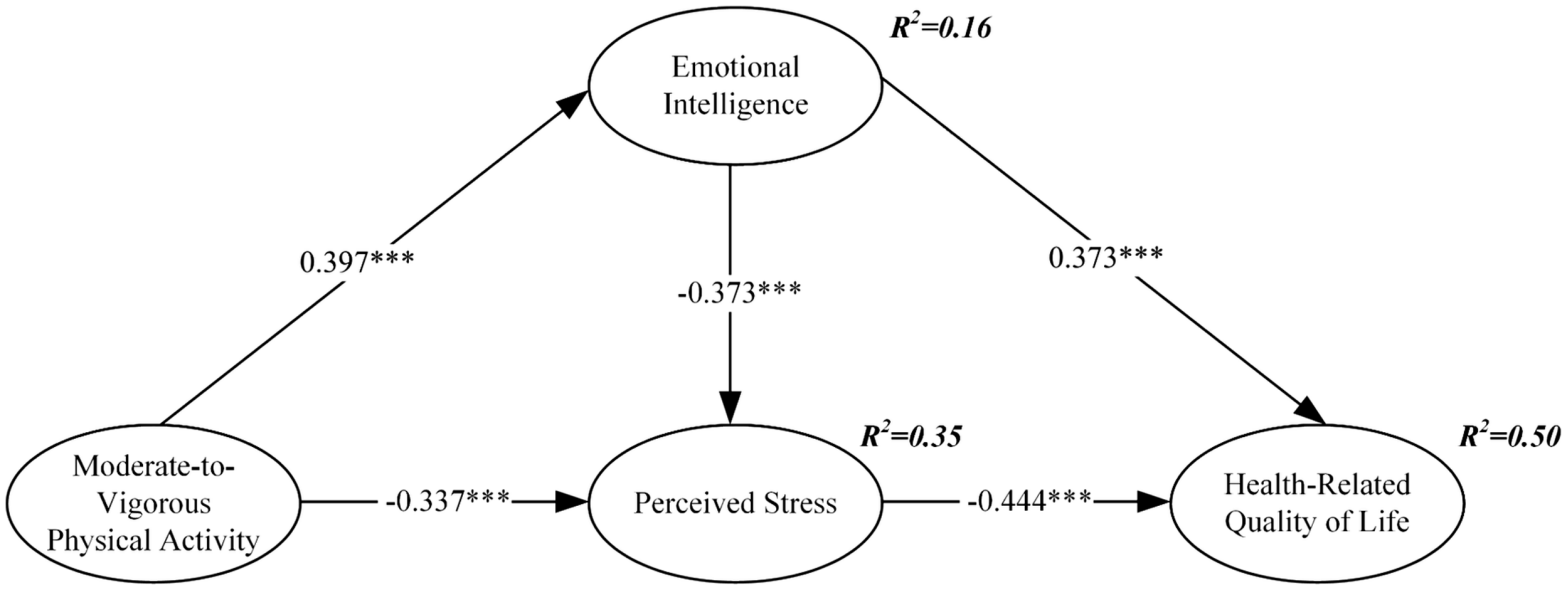
Path model: relationships among variables. ***p < 0.001.

As shown in Figure 2, MVPA had a significant positive direct association with emotional intelligence (*β* = 0.397, *p* < 0.001) and a significant negative direct relationship with perceived stress (*β* = −0.337, *p* < 0.001). Emotional intelligence was significantly negatively related to perceived stress (*β* = −0.373, *p* < 0.001) and positively related to HRQOL (*β* = 0.373, *p* < 0.001). Additionally, perceived stress had a significant negative direct correlation with HRQOL (*β* = −0.444, *p* < 0.001).

As shown in Table 4, the mediating effects were examined using bootstrap estimation with 5,000 resamples and 95% bias-corrected confidence intervals. The results indicate that emotional intelligence mediates the relationship between MVPA and perceived stress [coefficient = −0.148, SE = 0.028, CI = (−0.210, −0.100), *p* < 0.001], supporting Hypothesis 1. Perceived stress mediates the relationship between emotional intelligence and HRQOL [coefficient = 0.165, SE = 0.034, CI = (0.110, 0.244), *p* < 0.001], supporting Hypothesis 2. The pathway through which MVPA affects HRQOL, mediated by emotional intelligence and perceived stress, has a coefficient of 0.363 [SE = 0.038, CI = (0.282, 0.431), *p* < 0.001], supporting Hypothesis 3.

**Table 4:** Standardized indirect effect.

	Point estimate	Product of coefficients		Bootstrapping		
				Bias-corrected 95% CI	Two-tailed significance	
		SE	Z	Lower	Upper	
MVPA → PS	−0.148	0.028	5.286	−0.210	−0.100	< 0.001
EI → HRQOL	0.165	0.034	4.853	0.110	0.244	< 0.001
MVPA → HRQOL	0.363	0.038	9.553	0.282	0.431	< 0.001

## 5 Discussion

### 5.1 Theoretical contributions

Firstly, existing research primarily explores the impact of different intensities of physical activity on adolescents’ physical health and the role of MVPA in intervening in mild mental disorders (Arslan et al., 2022). Although previous studies have confirmed the positive impact of children and adolescents participating in physical activity on their HRQOL levels, there is still a lack of empirical research on how the intensity of physical activity affects adolescents’ HRQOL (Marker et al., 2018). Our study shifts the focus to examining how MVPA affects adolescents’ HRQOL at the cognitive level. This detailed perspective adds depth to the existing theoretical research in this field. Moreover, since previous studies indicate that the current generation of adolescents shows lower HRQOL levels compared to the previous generation, our study places greater emphasis on HRQOL levels during adolescence (Patton et al., 2016).

At the same time, even during major stress events like the COVID-19 pandemic, maintaining a high level of physical activity can help adolescents alleviate symptoms of insomnia, anxiety, and depression, thereby preserving their psychological and mental health (Chi et al., 2021). Our study found that MVPA has a significant inhibitory effect on perceived stress, a psychological issue. In fact, moderate-to-vigorous physical activities, such as various forms of high-intensity interval training (HIIT), can positively influence adolescents’ cognitive performance (attention, executive function, abstract reasoning, etc.) and mental health levels (Alves et al., 2021). Given that subjective cognitive mental health is a key subset for measuring HRQOL, researchers believe that understanding the cognitive impact of MVPA on HRQOL can help identify methods to adjust contemporary adolescents’ HRQOL levels.

Additionally, our study finds that the HRQOL level of Chinese adolescents is concerning, further validating previous research (Qin et al., 2021). This can be attributed to the numerous challenges Chinese adolescents face in academic progress and social interactions (Zeng et al., 2022), which have been exacerbated by the prolonged social isolation resulting from the recent COVID-19 pandemic (Magson et al., 2020). These factors further jeopardize the already fragile HRQOL levels of adolescents. Furthermore, the study points out that overweight and obesity issues are prevalent among today’s adolescents. MVPA can stimulate various physiological changes, enhance personal functions, and reduce perceived stress related to body image issues, such as obesity and overweight, thereby improving mental, physical health, and HRQOL levels.

The study also emphasizes that emotional intelligence is a valuable psychological resource that plays a positive role in resisting negative psychological states (Acebes-Sánchez et al., 2019). Consistent with previous studies, our research indicates that MVPA can influence adolescents’ HRQOL levels together with emotional intelligence (Ubago-Jiménez et al., 2021). However, this study further highlights that MVPA can positively impact adolescents’ HRQOL by inhibiting perceived stress. Additionally, our research innovatively explores the mediating roles of emotional intelligence and perceived stress in the relationship between MVPA and HRQOL. The results show that emotional intelligence serves as a notable positive mediator, while perceived stress acts as a negative mediator (see Figure 2). This supports the findings of Racic et al. (2017). These variables collectively explain 50% of the variance in HRQOL, with emotional intelligence having a greater inhibitory effect on perceived stress compared to MVPA.

Our study introduces an effective approach to investigating the link between MVPA and HRQOL, exploring the cognitive impacts of adolescents’ participation in MVPA in daily life. It demonstrates the synergistic inhibitory effects of MVPA and emotional intelligence on perceived stress, thereby expanding the scope of research on cognitive-level interventions.

### 5.2 Practical implications

We selected Hunan, Hubei, and Henan provinces, which form the central region of China, as the sources of the survey sample. These provinces are located in the inland central area of China, characterized by relatively balanced economic development, a large population, and a high level of education among adolescents. The investigation of samples from these three provinces provides a meaningful reference for studying the relationship between adolescents’ MVPA and HRQOL in other provinces of China. However, considering China’s unique social and cultural context, education system, and large population, which results in greater academic pressure and more acute HRQOL issues among adolescents (Qin et al., 2021; Zeng et al., 2022), the findings of our study also have a certain level of uniqueness.

This study suggests that MVPA positively affects adolescents’ emotional intelligence and negatively impacts perceived stress, highlighting the indirect relationship between MVPA and HRQOL levels in adolescents. To support adolescents’ engagement in MVPA, families, schools, and communities should provide both material and emotional support. Recognizing that promoting MVPA among adolescents is not a short-term task but a long-term commitment, this study provides practical guidance from short-, medium-, and long-term perspectives.

In the short term, parents should focus on their children’s extracurricular activities, actively participating in outdoor physical activities with them or encouraging them to join sports clubs during their free time. Limiting screen time while ensuring adequate sleep can promote more MVPA, thus improving adolescents’ HRQOL (Parker et al., 2022). Schools and communities should enhance both the quantity and quality of sports facilities and implement effective management mechanisms to ensure that students can easily participate in various sports activities.

In the medium term, schools should actively respond to China’s after-school physical education policies and adjust physical education classes based on students’ interests. This includes adapting curriculum content and teaching methods and encouraging physical education teachers to innovate to make classes more engaging. Additionally, schools should foster a culture of physical exercise throughout the institution by organizing various sports-related activities and mobilizing resources from parents, communities, and other stakeholders to create a supportive environment for students to engage in physical activities.

From a long-term perspective, schools should establish and improve sports management mechanisms to lay the foundation for lifelong physical activity among students. This includes developing clear plans and policies for the development of school sports, hiring professional physical education teachers and administrators, and ensuring the sustainability of sports programs. As a bridge between schools and families, communities should actively facilitate coordination between the two, forming a tripartite structure that involves families, schools, and communities working together. At the same time, we must build on the short- and medium-term efforts of families, schools, and communities to create a long-term positive cycle that encourages adolescents to continue participating in MVPA.

In today’s rapidly changing society, adolescents’ HRQOL faces various threats, including challenges brought by the evolving social environment (Panter-Brick et al., 2009; Lam and Peng, 2010; Parker et al., 2016). Additionally, the increasing academic burden continuously adds to adolescents’ perceived stress (Reverte-Villarroya et al., 2021; Zeng et al., 2022). According to the results of this study, adolescents enhance their emotional intelligence through MVPA, successfully alleviating perceived stress, and establishing a positive correlation with their HRQOL. This is because MVPA fosters adolescents’ self-identity through competitive sports activities and provides opportunities for collaboration with teammates, which helps counteract the social isolation caused by prolonged social distancing during the pandemic, thereby enhancing their emotional intelligence (Ubago-Jiménez et al., 2019). Simultaneously, adolescents release accumulated perceived stress from academics, family, and social relationships through MVPA. In this process, they temporarily disengage from the complexities of academic, family, and social relationships, immerse themselves in sports activities, and achieve short-term but effective relaxation, ultimately contributing to improved HRQOL levels. Additionally, adolescents themselves should actively embrace the triangle of physical activities built by the short-term efforts and long-term cycles of families, schools, and society. They should take advantage of available sports facilities, participate in physical activities, enjoy the sports environment, and achieve the goal of enhancing their MVPA.

However, despite the numerous documented benefits of MVPA in enhancing HRQOL, the actual participation rate, frequency, and intensity of MVPA among adolescents remain suboptimal. This is mainly due to the lack of awareness among society, schools, and parents about the importance of MVPA for adolescents’ HRQOL, coupled with an excessive focus on academic performance that forces Chinese adolescents to devote significant time and energy to studying (Liang et al., 2023). Moreover, the increasing use of smart mobile devices among adolescents in recent years has led some to escape reality through the internet when facing high perceived stress in real life, further encroaching on time that could be spent on physical activities.

Adjusting adolescents’ HRQOL levels is a long-term and continuous process. This study not only elucidates the intrinsic mechanisms between MVPA and HRQOL during adolescence but also encourages more adolescents to participate in MVPA to gain opportunities for peer competition and cooperation. Additionally, the study encourages adolescents to develop one or more sports hobbies and engage in them consistently, thereby resisting academic pressure and the temptations of electronic devices, ultimately promoting positive HRQOL levels.

### 5.3 Limitations

Firstly, our study selected data sources from three provinces in the central region of China. While this approach reduced the complexity of data collection and provided convenience, it must be acknowledged that this choice might slightly impact the representativeness of the sample. To address this limitation, future research in this field could consider expanding the sample sources. By including data from more diverse regions, researchers can enhance the geographic coverage and improve the representativeness of the sample, providing a more comprehensive understanding of the topic.

Secondly, our study primarily focused on investigating two cognitive mediators in the relationship between MVPA and HRQOL. Although this approach provided valuable insights, there remains room for future researchers to delve deeper into this relationship. Exploring additional mediating variables could elucidate the complex interactions between MVPA and HRQOL more thoroughly.

Thirdly, due to the limitations of our actual research capabilities, this study relies on adolescents’ self-reported measurements of MVPA. Self-reports may involve recall bias and inaccuracies. Future research should consider using wearable activity monitors to obtain more objective and accurate data on exercise intensity. This will improve the reliability of the data and provide a more precise understanding of adolescents’ MVPA levels.

Lastly, our study employed a cross-sectional method, lacking a temporal dimension. To further advance research in this area, future studies could adopt longitudinal research methods. This approach would offer valuable insights into the dynamic relationship between MVPA and HRQOL over time, helping researchers capture trends and changes.

By addressing these limitations and exploring new research avenues, future studies can build on our findings to provide a more nuanced and comprehensive understanding of how MVPA influences adolescents’ HRQOL.

## 6 Conclusion

To achieve the research objectives, this study validated the positive role of MVPA in regulating HRQOL among adolescents. Notably, emotional intelligence and perceived stress emerged as mediating factors in the impact of MVPA on psychological health. Additionally, MVPA and emotional intelligence jointly inhibited the development of perceived stress. The enhancement of emotional intelligence through MVPA proved more effective in reducing perceived stress, thereby promoting overall HRQOL among adolescents. Therefore, this study recommends moving away from the prevalent exam-oriented culture and encourages increased attention to adolescents’ HRQOL issues.

To create an environment in Chinese society that supports adolescents’ participation in MVPA and improves their MVPA levels, families, schools, and communities must form a triangular network of physical activity support from short-term efforts, to mid-term maintenance, and long-term cycling. This includes providing adolescents with emotional support from their families, material resources from the community, and institutional support from schools. For example, parents can spend more time engaging in physical activities with their children, communities can update and maintain facilities and equipment for youth sports activities, and schools can actively implement the national after-school physical education service policies.

## Data Availability

The raw data supporting the conclusions of this article will be made available by the authors, without undue reservation.

## References

[ref1] Acebes-Sánchez J. Diez-Vega I. Esteban-Gonzalo S. Rodriguez-Romo G. (2019). Physical activity and emotional intelligence among undergraduate students: a correlational study. BMC Public Health 19:1241. doi: 10.1186/s12889-019-7576-5, PMID: 31500593 PMC6734231

[ref2] Alves A. R. Dias R. Neiva H. P. Marinho D. A. Marques M. C. Sousa A. C.. (2021). High-intensity interval training upon cognitive and psychological outcomes in youth: a systematic review. Int. J. Environ. Res. Public Health 18:5344. doi: 10.3390/ijerph18105344, PMID: 34067861 PMC8157174

[ref3] Arslan G. Yıldırım M. Karataş Z. Kabasakal Z. Kılınç M. (2022). Meaningful living to promote complete mental health among university students in the context of the COVID-19 pandemic. Int. J. Ment. Health Addict. 20, 930–942. doi: 10.1007/s11469-020-00416-8, PMID: 33169074 PMC7609355

[ref4] Bermejo-Cantarero A. Álvarez-Bueno C. Martínez-Vizcaino V. Redondo-Tébar A. Pozuelo-Carrascosa D. P. Sánchez-López M. (2021). Relationship between both cardiorespiratory and muscular fitness and health-related quality of life in children and adolescents: a systematic review and meta-analysis of observational studies. Health. Qual. Life Out. 19:127. doi: 10.1186/s12955-021-01766-0, PMID: 33882937 PMC8059195

[ref5] Best O. Ban S. (2021). Adolescence: physical changes and neurological development. Br. J. Nurs. 30, 272–275. doi: 10.12968/bjon.2021.30.5.272 33733842

[ref6] Bisegger C. Cloetta B. von Bisegger U. Abel T. Ravens-Sieberer U. The European Kidscreen group (2005). Health-related quality of life: gender differences in childhood and adolescence. Soz. Praventiv. Med. 50, 281–291. doi: 10.1007/s00038-005-4094-2 16300172

[ref7] Blomster J. I. Chow C. K. Zoungas S. Woodward M. Patel A. Poulter N. R.. (2013). The influence of physical activity on vascular complications and mortality in patients with type 2 diabetes mellitus. Diabetes Obes. Metab. 15, 1008–1012. doi: 10.1111/dom.12122, PMID: 23675676

[ref8] Boraita R. J. Ibort E. G. Torres J. M. D. Alsina D. A. (2020). Gender differences relating to lifestyle habits and health-related quality of life of adolescents. Child Indic. Res. 13, 1937–1951. doi: 10.1007/s12187-020-09728-6

[ref9] Cejudo J. Rodrigo-Ruiz D. López-Delgado M. L. Losada L. (2018). Emotional intelligence and its relationship with levels of social anxiety and stress in adolescents. Int. J. Environ. Res. Public Health 15:1073. doi: 10.3390/ijerph15061073, PMID: 29799465 PMC6024952

[ref10] Chang Y. K. Labban J. Gapin J. J. Etnier J. L. (2012). The effects of acute exercise on cognitive performance: a meta-analysis. Brain Res. 1453, 87–101. doi: 10.1016/j.brainres.2012.02.068 22480735

[ref11] Chi X. Liang K. Chen S. T. Huang Q. Huang L. Yu Q.. (2021). Mental health problems among Chinese adolescents during the COVID-19: the importance of nutrition and physical activity. Int. J. Clin. Hlth. Psyc. 21:100218. doi: 10.1016/j.ijchp.2020.100218 PMC7759093 33391373

[ref12] Cohen S. Kamarck T. Mermelstein R. (1983). A global measure of perceived stress. J. Health Soc. Behav., 24, 385–396. doi: 10.2307/2136404 6668417

[ref13] Corell M. Friberg P. Löfstedt P. Petzold M. Chen Y. (2021). Subjective health complaints in early adolescence reflect stress: a study among adolescents in Western Sweden. Scand. J. Public Health 50, 516–523. doi: 10.1177/14034948211008555, PMID: 33863257 PMC9152601

[ref14] Crosby R. D. Kolotkin R. L. Williams G. R. (2003). Defining clinically meaningful change in health-related quality of life. J. Clin. Epidemiol. 56, 395–407. doi: 10.1016/S0895-4356(03)00044-1 12812812

[ref15] de Belvis A. G. Avolio M. Spagnolo A. Damiani G. Sicuro L. Cicchetti A.. (2008). Factors associated with health-related quality of life: the role of social relationships among the elderly in an Italian region. Public Health 122, 784–793. doi: 10.1016/j.puhe.2007.08.018, PMID: 18374375

[ref16] Dhillon S. Videla-Nash G. Foussias G. Segal Z. V. Zakzanis K. K. (2020). On the nature of objective and perceived cognitive impairments in depressive symptoms and real-world functioning in young adults. Psychiatry Res. 287:112932. doi: 10.1016/j.psychres.2020.112932, PMID: 32272334

[ref17] Dubey V. P. Kievišienė J. Rauckiene-Michealsson A. Norkiene S. Razbadauskas A. Agostinis-Sobrinho C. (2022). Bullying and health related quality of life among adolescents—a systematic review. Children 9:766. doi: 10.3390/children9060766, PMID: 35740703 PMC9222044

[ref18] Estrada M. Monferrer D. Rodríguez A. Moliner M. Á. (2021). Does emotional intelligence influence academic performance? The role of compassion and engagement in education for sustainable development. Sustain. For. 13:1721. doi: 10.3390/su13041721

[ref19] Fornell C. Larcker D. F. (1981). Evaluating structural equation models with unobservable variables and measurement error. J. Mark. Res. 18, 39–50. doi: 10.1177/002224378101800104

[ref20] Freak-Poli R. Ryan J. Tran T. Owen A. McHugh Power J. Berk M.. (2022). Social isolation, social support and loneliness as independent concepts, and their relationship with health-related quality of life among older women. Aging Ment. Health 26, 1335–1344. doi: 10.1080/13607863.2021.1940097, PMID: 34219569

[ref21] Frisén A. (2007). Measuring health-related quality of life in adolescence. Acta Paediatr. 96, 963–968. doi: 10.1111/j.1651-2227.2007.00333.x 17498184

[ref22] Gerber M. Ludyga S. Mücke M. Colledge F. Brand S. Pühse U. (2017). Low vigorous physical activity is associated with increased adrenocortical reactivity to psychosocial stress in students with high stress perceptions. Psychoneuroendocrinology 80, 104–113. doi: 10.1016/j.psyneuen.2017.03.004 28324699

[ref23] Gomes A. C. Rebelo M. A. B. de Queiroz A. C. de Queiroz Herkrath A. P. C. Herkrath F. J. Rebelo Vieira J. M.. (2020). Socioeconomic status, social support, oral health beliefs, psychosocial factors, health behaviours and health-related quality of life in adolescents. Qual. Life Res. 29, 141–151. doi: 10.1007/s11136-019-02279-6, PMID: 31468278

[ref24] González-Cabrera J. Machimbarrena J. M. Fernández-González L. Prieto-Fidalgo Á. Vergara-Moragues E. Calvete E. (2021). Health-related quality of life and cumulative psychosocial risks in adolescents. Youth Soc. 53, 636–653. doi: 10.1177/0044118x19879461

[ref25] Grant K. E. Compas B. E. Thurm A. E. McMahon S. D. Gipson P. Y. (2004). Stressors and child and adolescent psychopathology: measurement issues and prospective effects. J. Clin. Child Adolesc. Psychol. 33, 412–425. doi: 10.1207/s15374424jccp3302_23, PMID: 15136206

[ref26] Gu N. (2022) Physical education and health curriculum standards for compulsory education (2022 edition) released - school physical education classes to become more interesting.

[ref27] Hale G. E. Colquhoun L. Lancastle D. Lewis N. Tyson P. J. (2021). Review: physical activity interventions for the mental health and well-being of adolescents – a systematic review. Child Adolesc. Ment. Health 26, 357–368. doi: 10.1111/camh.12485, PMID: 34105239

[ref28] Hampel P. Petermann F. (2006). Perceived stress, coping, and adjustment in adolescents. J. Adolesc. Health 38, 409–415. doi: 10.1016/j.jadohealth.2005.02.014 16549302

[ref29] Haraldstad K. Christophersen K. A. Eide H. Nativg G. K. Helseth S. (2011). Predictors of health-related quality of life in a sample of children and adolescents: a school survey. J. Clin. Nurs. 20, 3048–3056. doi: 10.1111/j.1365-2702.2010.03693.x, PMID: 21320221

[ref30] Herbert C. Meixner F. Wiebking C. Gilg V. (2020). Regular physical activity, short-term exercise, mental health, and well-being among university students: the results of an online and a laboratory study. Front. Psychol. 11:509. doi: 10.3389/fpsyg.2020.00509, PMID: 32528333 PMC7264390

[ref31] Hollis J. L. Sutherland R. Williams A. J. Campbell E. Nathan N. Wolfenden L.. (2017). A systematic review and meta-analysis of moderate-to-vigorous physical activity levels in secondary school physical education lessons. Int. J. Behav. Nutr. Phys. Act. 14:52. doi: 10.1186/s12966-017-0504-0, PMID: 28438171 PMC5402678

[ref32] Jackson C. A. Henderson M. Frank J. W. Haw S. J. (2012). An overview of prevention of multiple risk behaviour in adolescence and young adulthood. J. Public Health 34, i31–i40. doi: 10.1093/pubmed/fdr113 22363029

[ref33] Jiménez Boraita R. Gargallo Ibort E. Dalmau Torres J. M. Arriscado Alsina D. (2024). Lifestyle habits, health indicators and sociodemographic factors associated with health-related quality of life and self-esteem in adolescents. Clin. Child Psychol. P. 29, 493–512. doi: 10.1177/13591045231200661, PMID: 37658652

[ref34] Jurakić D. Pedišić Ž. Greblo Z. (2010). Physical activity in different domains and health-related quality of life: a population-based study. Qual. Life Res. 19, 1303–1309. doi: 10.1007/s11136-010-9705-6 20632116

[ref35] Kaur T. Agarwal S. (2022). Correlation between perceived stress, physical fitness and health related quality of life in young adults. J. Clin. Diagnostic Res. 16. doi: 10.7860/JCDR/2022/56794.16418

[ref36] Knaeps S. Lefevre J. Wijtzes A. Charlier R. Mertens E. Bourgois J. G. (2016). Independent associations between sedentary time, moderate-to-vigorous physical activity, cardiorespiratory fitness and cardio-metabolic health: a cross-sectional study. PLoS One 11:e0160166. doi: 10.1371/journal.pone.0160166, PMID: 27463377 PMC4963092

[ref37] la Du T. J. Tanaka J. S. (1989). Influence of sample size, estimation method, and model specification on goodness-of-fit assessments in structural equation models. J. Appl. Psychol. 74, 625–635. doi: 10.1037/0021-9010.74.4.625

[ref38] Lam L. T. Peng Z. W. (2010). Effect of pathological use of the internet on adolescent mental health. Arch. Pediatr. Adolesc. Med. 164, 901–906. doi: 10.1001/archpediatrics.2010.159 20679157

[ref39] Law K. S. Wong C.-S. Song L. J. (2004). The construct and criterion validity of emotional intelligence and its potential utility for management studies. J. Appl. Psychol. 89, 483–496. doi: 10.1037/0021-9010.89.3.483, PMID: 15161407

[ref40] Lee F. S. Heimer H. Giedd J. N. Lein E. S. Šestan N. Weinberger D. R.. (2014). Adolescent mental health—opportunity and obligation. Science 346, 547–549. doi: 10.1126/science.1260497, PMID: 25359951 PMC5069680

[ref41] Liang K. Chen S. Chi X. (2023). Differential associations between meeting 24-hour movement guidelines with mental wellbeing and mental illness among Chinese adolescents. J. Adolesc. Health 72, 658–666. doi: 10.1016/j.jadohealth.2022.11.231 36599758

[ref42] Lindholdt L. Labriola M. Andersen J. H. Kjeldsen M. M. Z. Obel C. Lund T. (2021). Perceived stress among adolescents as a marker for future mental disorders: a prospective cohort study. Scand. J. Public Health 50, 412–417. doi: 10.1177/1403494821993719, PMID: 33641501 PMC9096581

[ref43] Liu J. J. W. Reed M. Girard T. A. (2017). Advancing resilience: an integrative, multi-system model of resilience. Pers. Indiv. Differ. 111, 111–118. doi: 10.1016/j.paid.2017.02.007

[ref44] Lueke N. A. Assar A. (2024). Poor sleep quality and reduced immune function among college students: perceived stress and depression as mediators. J. Am. Coll. Heal. 72, 1112–1119. doi: 10.1080/07448481.2022.2068350 35549834

[ref45] Luo S. Feng X. Lin L. Li J. Chen W. Guo V. Y. (2024). Association of adverse and positive childhood experiences with health-related quality of life in adolescents. Public Health 228, 92–99. doi: 10.1016/j.puhe.2024.01.006 38340507

[ref46] Magson N. R. Freeman J. Y. A. Rapee R. M. Richardson C. E. Oar E. L. Fardouly J. (2020). Risk and protective factors for prospective changes in adolescent mental health during the COVID-19 pandemic. J. Youth Adolesc. 50, 44–57. doi: 10.1007/s10964-020-01332-9, PMID: 33108542 PMC7590912

[ref47] Mahindru A. Patil P. Agrawal V. (2023). Role of physical activity on mental health and well-being: a review. Cureus 15:e33475. doi: 10.7759/cureus.33475, PMID: 36756008 PMC9902068

[ref48] Malinauskas R. Malinauskiene V. (2020). The relationship between emotional intelligence and psychological well-being among male university students: the mediating role of perceived social support and perceived stress. Int. J. Environ. Res. Public Health 17:1605. doi: 10.3390/ijerph17051605, PMID: 32131478 PMC7084724

[ref49] Manh Than H. Minh Nong V. Trung Nguyen C. Phu Dong K. Ngo H. T. Thu Doan T.. (2020). Mental health and health-related quality-of-life outcomes among frontline health workers during the peak of COVID-19 outbreak in Vietnam: a cross-sectional study. Risk Manag. Healthc. P 13, 2927–2936. doi: 10.2147/RMHP.S280749, PMID: 33324126 PMC7733435

[ref50] Marker A. M. Steele R. G. Noser A. E. (2018). Physical activity and health-related quality of life in children and adolescents: a systematic review and meta-analysis. Health Psychol. 37, 893–903. doi: 10.1037/hea0000653 30234348

[ref51] Martorell-Poveda M.-A. Martinez-Hernáez A. Carceller-Maicas N. Correa-Urquiza M. (2015). Self-care strategies for emotional distress among young adults in Catalonia: a qualitative study. Int. J. Ment. Health Syst 9:9. doi: 10.1186/s13033-015-0001-2, PMID: 25788975 PMC4363191

[ref52] Mikkelsen H. T. Haraldstad K. Helseth S. Skarstein S. Småstuen M. C. Rohde G. (2020). Health-related quality of life is strongly associated with self-efficacy, self-esteem, loneliness, and stress in 14–15-year-old adolescents: a cross-sectional study. Health Qual. Life Outcomes 18:352. doi: 10.1186/s12955-020-01585-9, PMID: 33138833 PMC7607747

[ref53] Mikkelsen K. Stojanovska L. Polenakovic M. Bosevski M. Apostolopoulos V. (2017). Exercise and mental health. Maturitas 106, 48–56. doi: 10.1016/j.maturitas.2017.09.003 29150166

[ref54] Moore G. F. Cox R. Evans R. E. Hallingberg B. Hawkins J. Littlecott H. J.. (2018). School, peer and family relationships and adolescent substance use, subjective wellbeing and mental health symptoms in Wales: a cross sectional study. Child Indic. Res. 11, 1951–1965. doi: 10.1007/s12187-017-9524-1 30524519 PMC6244918

[ref55] Mossholder K. W. Bennett N. Kemery E. R. Wesolowski M. A. (1998). Relationships between bases of Power and work reactions: the mediational role of procedural justice. J. Manag. 24, 533–552. doi: 10.1177/014920639802400404

[ref56] Nader P. R. Bradley R. H. Houts R. M. McRitchie S. O’Briendo M. (2008). Moderate-to-vigorous physical activity from ages 9 to 15 years. JAMA 300, 295–305. doi: 10.1001/jama.300.3.295 18632544

[ref57] Nagata J. M. Cortez C. A. Dooley E. E. Iyer P. Ganson K. T. Pettee Gabriel K. (2022). Moderate-to-vigorous intensity physical activity among adolescents in the USA during the COVID-19 pandemic. Prev. Med. Rep. 25:101685. doi: 10.1016/j.pmedr.2021.101685, PMID: 35004134 PMC8719022

[ref58] Nakagawa T. Koan I. Chen C. Matsubara T. Hagiwara K. Lei H.. (2020). Regular moderate- to vigorous-intensity physical activity rather Than walking is associated with enhanced cognitive functions and mental health in young adults. Int. J. Environ. Res. Public Health 17:614. doi: 10.3390/ijerph17020614, PMID: 31963639 PMC7014044

[ref59] Nelis D. Quoidbach J. Mikolajczak M. Hansenne M. (2009). Increasing emotional intelligence: (how) is it possible? Pers. Individ. Differ. 47, 36–41. doi: 10.1016/j.paid.2009.01.046

[ref60] Nik-Azin A. Shairi M. R. Naeinian M. R. Sadeghpour A. (2014). The health-related quality of life index KIDSCREEN-10: confirmatory factor analysis, convergent validity and reliability in a sample of Iranian students. Child Indic. Res. 7, 407–420. doi: 10.1007/s12187-013-9216-4

[ref61] Panter-Brick C. Eggerman M. Gonzalez V. Safdar S. (2009). Violence, suffering, and mental health in Afghanistan: a school-based survey. Lancet 374, 807–816. doi: 10.1016/S0140-6736(09)61080-1 19699514 PMC2748901

[ref62] Parker P. D. Jerrim J. Anders J. (2016). What effect did the global financial crisis have upon youth wellbeing? Evidence from four Australian cohorts. Dev. Psychol. 52, 640–651. doi: 10.1037/dev0000092, PMID: 26854968 PMC4819495

[ref63] Parker A. G. Trott E. Bourke M. Klepac Pogrmilovic B. Dadswell K. Craike M.. (2022). Young people’s attitudes towards integrating physical activity as part of mental health treatment: a cross-sectional study in youth mental health services. Early Interv. Psychiatry 16, 518–526. doi: 10.1111/eip.13189 34312996

[ref64] Pascoe M. Bailey A. P. Craike M. Carter T. Patten R. Stepto N.. (2020). Physical activity and exercise in youth mental health promotion: a scoping review. BMJ Open Sport Exerc. Med. 6:e000677. doi: 10.1136/bmjsem-2019-000677, PMID: 32095272 PMC7010991

[ref65] Patton G. C. Sawyer S. M. Santelli J. S. Ross D. A. Afifi R. Allen N. B.. (2016). Our future: a lancet commission on adolescent health and wellbeing. Lancet 387, 2423–2478. doi: 10.1016/S0140-6736(16)00579-1, PMID: 27174304 PMC5832967

[ref66] Petrides K. V. Mikolajczak M. Mavroveli S. Sanchez-Ruiz M. J. Furnham A. Pérez-González J. C. (2016). Developments in trait emotional intelligence research. Emot. Rev. 8, 335–341. doi: 10.1177/1754073916650493

[ref67] Pfeifer J. H. Allen N. B. (2021). Puberty initiates cascading relationships between neurodevelopmental, social, and internalizing processes across adolescence. Biol. Psychiatry 89, 99–108. doi: 10.1016/j.biopsych.2020.09.002, PMID: 33334434 PMC8494463

[ref68] Piekarska J. (2020). Determinants of perceived stress in adolescence: the role of personality traits, emotional abilities, trait emotional intelligence, self-efficacy, and self-esteem. Adv. Cogn. Psychol. 16, 309–320. doi: 10.5709/acp-0305-z, PMID: 33532008 PMC7839256

[ref69] Pinquart M. (2020). Health-related quality of life of young people with and without chronic conditions. J. Pediatr. Psychol. 45, 780–792. doi: 10.1093/jpepsy/jsaa052 32642762

[ref70] Piqueras J. A. Kuhne W. Vera-Villarroel P. van Straten A. Cuijpers P. (2011). Happiness and health behaviours in Chilean college students: a cross-sectional survey. BMC Public Health 11:443. doi: 10.1186/1471-2458-11-443, PMID: 21649907 PMC3125376

[ref71] Pollatos O. Georgiou E. Kobel S. Schreiber A. Dreyhaupt J. Steinacker J. M. (2020). Trait-based emotional intelligence, body image dissatisfaction, and HRQoL in children. Front. Psych. 10:973. doi: 10.3389/fpsyt.2019.00973 PMC6990369 32038322

[ref72] Puertas Molero P. González Valero G. Sánchez Zafra M. (2017). Influencia de la práctica físico deportiva sobre la Inteligencia Emocional de los estudiantes: Una revisión sistemática. ESHPA. 1, 10–24.

[ref73] Qin Z. Wang N. Ware R. S. Sha Y. Xu F. (2021). Lifestyle-related behaviors and health-related quality of life among children and adolescents in China. Health Qual. Life Outcomes 19:8. doi: 10.1186/s12955-020-01657-w, PMID: 33407589 PMC7788787

[ref74] Racic M. Todorovic R. Ivkovic N. Masic S. Joksimovic B. Kulic M. (2017). Self- perceived stress in relation to anxiety, depression and health-related quality of life among health professions students: a cross-sectional study from Bosnia and Herzegovina %J Slovenian journal of public health. Slovenian J. Public Health 56, 251–259. doi: 10.1515/sjph-2017-0034, PMID: 29062400 PMC5639815

[ref75] Rapee R. M. Magson N. R. Forbes M. K. Richardson C. E. Johnco C. J. Oar E. L.. (2022). Risk for social anxiety in early adolescence: longitudinal impact of pubertal development, appearance comparisons, and peer connections. Behav. Res. Ther. 154:104126. doi: 10.1016/j.brat.2022.104126, PMID: 35642989

[ref76] Ravens-Sieberer U. Erhart M. Rajmil L. Herdman M. Auquier P. Bruil J.. (2010). Reliability, construct and criterion validity of the KIDSCREEN-10 score: a short measure for children and adolescents’ well-being and health-related quality of life. Qual. Life Res. 19, 1487–1500. doi: 10.1007/s11136-010-9706-5, PMID: 20668950 PMC2977059

[ref77] Reverte-Villarroya S. Ortega L. Raigal-Aran L. Sauras-Colon E. Ricoma-Muntane R. Ballester-Ferrando D.. (2021). Psychological well-being in nursing students: a multicentric, cross-sectional study. Int. J. Environ. Res. Public Health 18:3020. doi: 10.3390/ijerph18063020, PMID: 33804156 PMC7999566

[ref78] Richardson S. Shaffer J. A. Falzon L. Krupka D. Davidson K. W. Edmondson D. (2012). Meta-analysis of perceived stress and its association with incident coronary heart disease. Am. J. Cardiol. 110, 1711–1716. doi: 10.1016/j.amjcard.2012.08.004, PMID: 22975465 PMC3511594

[ref79] Ruiz-Aranda D. Extremera N. Pineda-Galán C. (2014). Emotional intelligence, life satisfaction and subjective happiness in female student health professionals: the mediating effect of perceived stress. J. Psychiatr. Ment. Health Nurs. 21, 106–113. doi: 10.1111/jpm.12052, PMID: 23578272

[ref80] Sallis J. F. Haskell W. L. Wood P. D. Fortmann S. P. Rogers T. Blair S. N.. (1985). Physical activity assessment methodology in the five-city PROJECT1. Am. J. Epidemiol. 121, 91–106. doi: 10.1093/oxfordjournals.aje.a113987, PMID: 3964995

[ref81] Salovey P. Mayer J. D. (1990). Emotional Intelligence. Imagin. Cogn. Pers. 9, 185–211. doi: 10.2190/dugg-p24e-52wk-6cdg

[ref82] Salovey P. Stroud L. R. Woolery A. Epel E. S. (2002). Perceived emotional intelligence, stress reactivity, and symptom reports: further explorations using the trait Meta-mood scale. Psychol. Health 17, 611–627. doi: 10.1080/08870440290025812

[ref83] Sánchez-Álvarez N. Extremera N. Fernández-Berrocal P. (2016). The relation between emotional intelligence and subjective well-being: a meta-analytic investigation. J. Posit. Psychol. 11, 276–285. doi: 10.1080/17439760.2015.1058968

[ref84] Sanchez-Ruiz M.-J. Tadros N. Khalaf T. Ego V. Eisenbeck N. Carreno D. F.. (2021). Trait emotional intelligence and wellbeing during the pandemic: the mediating role of meaning-centered coping. Front. Psychol. 12:648401. doi: 10.3389/fpsyg.2021.648401, PMID: 34054650 PMC8155707

[ref85] Sawyer S. M. Afifi R. A. Bearinger L. H. Blakemore S. J. Dick B. Ezeh A. C.. (2012). Adolescence: a foundation for future health. Lancet 379, 1630–1640. doi: 10.1016/S0140-6736(12)60072-5 22538178

[ref86] Schiffrin H. H. Nelson S. K. (2010). Stressed and happy? Investigating the relationship between happiness and perceived stress. J. Happiness Stud. 11, 33–39. doi: 10.1007/s10902-008-9104-7

[ref87] Siqueira Reis R. Ferreira Hino A. A. Romélio Rodriguez Añez C. (2010). Perceived stress scale:reliability and validity study in Brazil. J. Health Psychol. 15, 107–114. doi: 10.1177/1359105309346343 20064889

[ref88] Skrede T. Stavnsbo M. Aadland E. Aadland K. N. Anderssen S. A. Resaland G. K.. (2017). Moderate-to-vigorous physical activity, but not sedentary time, predicts changes in cardiometabolic risk factors in 10-y-old children: the active smarter kids study. Am. J. Clin. Nutr. 105, 1391–1398. doi: 10.3945/ajcn.116.150540 28381476

[ref89] Soares Filho L. C. Batista R. F. L. Cardoso V. C. Simões V. M. F. Santos A. M. Coelho S. J. D. D. A. C.. (2021). Body image dissatisfaction and symptoms of depression disorder in adolescents. Braz. J. Med. Biol. Res. 54. doi: 10.1590/1414-431X202010397 PMC7727113 33295537

[ref90] Tajik E. Abd Latiff L. Adznam S. N. Awang H. Siew C. Y. Bakar A. A. (2017). A study on level of physical activity, depression, anxiety and stress symptoms among adolescents. J. Sport Exerc. Psychol. 57, 1382–1387. doi: 10.23736/S0022-4707.16.06658-5 28004901

[ref91] Tang Y. Jing L. Liu Y. Wang H. (2023). Association of mindfulness on state-trait anxiety in choking-susceptible athletes: mediating roles of resilience and perceived stress. Front. Psychol. 14:1232929. doi: 10.3389/fpsyg.2023.1232929 37711325 PMC10497761

[ref92] Tapia-Serrano M. A. Sevil-Serrano J. Sánchez-Miguel P. A. López-Gil J. F. Tremblay M. S. García-Hermoso A. (2022). Prevalence of meeting 24-hour movement guidelines from pre-school to adolescence: a systematic review and meta-analysis including 387,437 participants and 23 countries. J. Sport Health Sci. 11, 427–437. doi: 10.1016/j.jshs.2022.01.005, PMID: 35066216 PMC9338333

[ref93] Tilga H. Kalajas-Tilga H. Hein V. Raudsepp L. Koka A. (2021). Perceived autonomy support from peers, parents, and physical education teachers as predictors of physical activity and health-related quality of life among adolescents—a one-year longitudinal study. Educ. Sci. 11:457. doi: 10.3390/educsci11090457

[ref94] Torrance G. W. J. (1987). Utility approach to measuring health-related quality of life. J. Chronic Dis. 40, 593–600. doi: 10.1016/0021-9681(87)90019-1 3298297

[ref95] Tremblay M. S. Carson V. Chaput J. P. Connor Gorber S. Dinh T. Duggan M.. (2016). Canadian 24-hour movement guidelines for children and youth: an integration of physical activity, sedentary behaviour, and sleep. Appl. Physiol. Nutr. Me. 41, S311–S327. doi: 10.1139/apnm-2016-0151 27306437

[ref96] Tsai C.-L. Chang Y. C. Pan C. Y. Wang T. C. Ukropec J. Ukropcová B. (2021). Acute effects of different exercise intensities on executive function and oculomotor performance in middle-aged and older adults: moderate-intensity continuous exercise vs. high-intensity interval exercise. Front. Aging Neurosci. 13:13. doi: 10.3389/fnagi.2021.743479, PMID: 34720993 PMC8548419

[ref97] Tseng T.-H. Chen H.-C. Wang L.-Y. Chien M.-Y. (2020). Effects of exercise training on sleep quality and heart rate variability in middle-aged and older adults with poor sleep quality: a randomized controlled trial. J. Clin. Sleep Med. 16, 1483–1492. doi: 10.5664/jcsm.8560 32394890 PMC7970583

[ref98] Ubago-Jiménez J. L. Cepero-González M. Martínez-Martínez A. Chacón-Borrego F. (2021). Linking emotional intelligence, physical activity and aggression among undergraduates. Int. J. Environ. Res. Public Health 18:12477. doi: 10.3390/ijerph182312477, PMID: 34886203 PMC8656989

[ref99] Ubago-Jiménez J. L. González-Valero G. Puertas-Molero P. García-Martínez I. (2019). Development of emotional intelligence through physical activity and sport practice. A systematic review. Int. J. Environ. Res. Public Health 9:44. doi: 10.3390/bs9040044, PMID: 31022863 PMC6523064

[ref100] van de Pas K. G. H. de Krom M. A. P. Winkens B. van Dielen F. M. H. Vreugdenhil A. C. E. (2023). Health-related quality of life in children and adolescents with overweight, obesity, and severe obesity: a cross-sectional study. Obes. Facts 16, 282–292. doi: 10.1159/000529560, PMID: 36758535 PMC10331158

[ref101] van Jaarsveld C. H. M. Fidler J. A. Steptoe A. Boniface D. Wardle J. (2009). Perceived stress and weight gain in adolescence: a longitudinal analysis. Obesity 17, 2155–2161. doi: 10.1038/oby.2009.183, PMID: 19521353

[ref102] Wang K. Li Y. Zhang T. Luo J. (2022). The relationship among college students’ physical exercise, self-efficacy, emotional intelligence, and subjective well-being. Int. J. Environ. Res. Public Health 19:11596. doi: 10.3390/ijerph191811596, PMID: 36141869 PMC9517190

[ref103] Wang H. Liu Y. Zhang S. Xu Z. Yang J. (2023). Investigating links between moderate-to-vigorous physical activity and self-rated health status in adolescents: the mediating roles of emotional intelligence and psychosocial stress. Children 10:1106. doi: 10.3390/children10071106 37508604 PMC10378217

[ref104] Wang K. Yang Y. Zhang T. Ouyang Y. Liu B. Luo J. (2020). The relationship between physical activity and emotional intelligence in college students: the mediating role of self-efficacy. Front. Psychol. 11:967. doi: 10.3389/fpsyg.2020.00967, PMID: 32581908 PMC7296084

[ref105] Ware J. E. Jr. J.E.J.A.O.P.M. and rehabilitation (2003). Conceptualization and measurement of health-related quality of life: comments on an evolving field. Arch. Phys. Med. Rehabil. 84, S43–S51. doi: 10.1053/apmr.2003.50246 12692771

[ref106] Wright L. J. Veldhuijzen van Zanten J. J. C. S. Williams S. E. (2023). Examining the associations between physical activity, self-esteem, perceived stress, and internalizing symptoms among older adolescents. J. Adolesc. 95, 1274–1287. doi: 10.1002/jad.12201, PMID: 37248071

[ref107] Wright L. J. Williams S. E. Veldhuijzen van Zanten J. J. C. S. (2021). Physical activity protects against the negative impact of coronavirus fear on adolescent mental health and well-being during the COVID-19 pandemic. Front. Psychol. 12:580511. doi: 10.3389/fpsyg.2021.580511, PMID: 33776827 PMC7990778

[ref108] Wu X. Y. Han L. H. Zhang J. H. Luo S. Hu J. W. Sun K. (2017). The influence of physical activity, sedentary behavior on health-related quality of life among the general population of children and adolescents: a systematic review. PLoS One 12:e0187668. doi: 10.1371/journal.pone.0187668, PMID: 29121640 PMC5679623

[ref109] Zeng J. Qiu N. Leitzelar B. N. Fu J. Wang Y. Liang F.. (2022). Parental Support Is Associated with Moderate to Vigorous Physical Activity among Chinese Adolescents through the Availability of Physical Activity Resources in the Home Environment and Autonomous Motivation. Children 9:1309. doi: 10.3390/children9091309, PMID: 36138618 PMC9498064

[ref110] Zhang Z. Wang T. Kuang J. Herold F. Ludyga S. Li J.. (2022). The roles of exercise tolerance and resilience in the effect of physical activity on emotional states among college students. Int. J. Clin. Health Psychol. 22:100312. doi: 10.1016/j.ijchp.2022.100312, PMID: 35712359 PMC9168153

[ref111] Zhou X. Li J. Jiang X. (2024). Effects of different types of exercise intensity on improving health-related physical fitness in children and adolescents: a systematic review. Sci. Rep. 14:14301. doi: 10.1038/s41598-024-64830-x, PMID: 38906965 PMC11192957

